# The outcomes of valve-in-valve TMVR for patients with mitral bioprosthetic valve dysfunction: a single-center retrospective study

**DOI:** 10.3389/fcvm.2026.1747077

**Published:** 2026-04-21

**Authors:** Tao Zhang, Yahua Li, Bin Lin, Li Li, Bowen Lian, Pengju Guo, Xiaoyan Zhao

**Affiliations:** 1 Department of Cardiovascular Surgery, The First Affiliated Hospital of Zhengzhou University, Zhengzhou, China; 2 Department of Interventional Radiology, The First Affiliated Hospital of Zhengzhou University, Zhengzhou, China; 3 Department of Cardiology, The First Affiliated Hospital of Zhengzhou University, Zhengzhou, China

**Keywords:** bioprosthetic valve dysfunction, TMVR, transapical, transseptal, valve-in-valve (VIV)

## Abstract

**Objective:**

To explore the clinical efficacy and follow-up results of the valve-in-valve transcatheter mitral valve replacement (TMVR) technique in patients with mitral bioprosthetic valve dysfunction.

**Methods:**

The medical data of patients with biological valve dysfunction after mitral valve replacement who underwent TMVR in our hospital from January 2019 to January 2024 were retrospectively collected. The echocardiography data, New York Heart Association (NYHA) grade, EuroQol visual analogue scale (EQ-VAS) score, and 6-minute walking distance before and after the operation were compared.

**Results:**

A total of 33 patients, 8 males and 25 females, with an average age of 70.70 ± 9.04 years, were included in this study. All 33 patients underwent TMVR surgery successfully. Seven patients underwent surgery via the atrial septal approach, and 26 patients underwent surgery via the apical approach. The success rate of the TMVR was 100.0%. One patient had a complication of cerebral infarction and eventually died 7 days after the operation. Compared with those prior to the operation, the NYHA grade, EQ-VAS score and the 6-minute walking distance were improved significantly at follow-up (all *P* < 0.05).

**Conclusion:**

Valve-in-valve TMVR is a feasible option for patients with degenerated bioprosthetic mitral valves. However, follow-up studies are still needed to determine the long-term effects.

## Introduction

Surgical valve replacement is an important method for treating moderate to severe mitral valve diseases. Artificial biological valves have the advantages of not requiring life-long anticoagulant medication and being more aligned with human haemodynamics (1, 2). However, owing to population ageing and the extension of the average life expectancy, valve deterioration has become a fatal shortcoming of biological valves (3, 4). Once the bivalve is damaged, the risk associated with a second valve replacement surgery under cardiopulmonary bypass is very high. Studies have shown that the mortality rate of a second thoracotomy can even reach 12.5% (3). Compared with that of the first mitral valve surgery, the 30-day mortality rate of repeated mitral valve surgeries is usually greater. Valve-in-valve transcatheter mitral valve replacement (TMVR) has gained considerable popularity and has currently been proven to be an effective alternative to surgical mitral valve replacement for high-risk patients (5, 6). The prognosis at 2 years after the operation is acceptable. In this study, we aimed to summarize the treatment experience of our centre and explore the clinical efficacy and follow-up results of the valve-in-valve TMVR technique in patients with mitral bioprosthetic valve dysfunction.

## Methods

**Figure 1:**
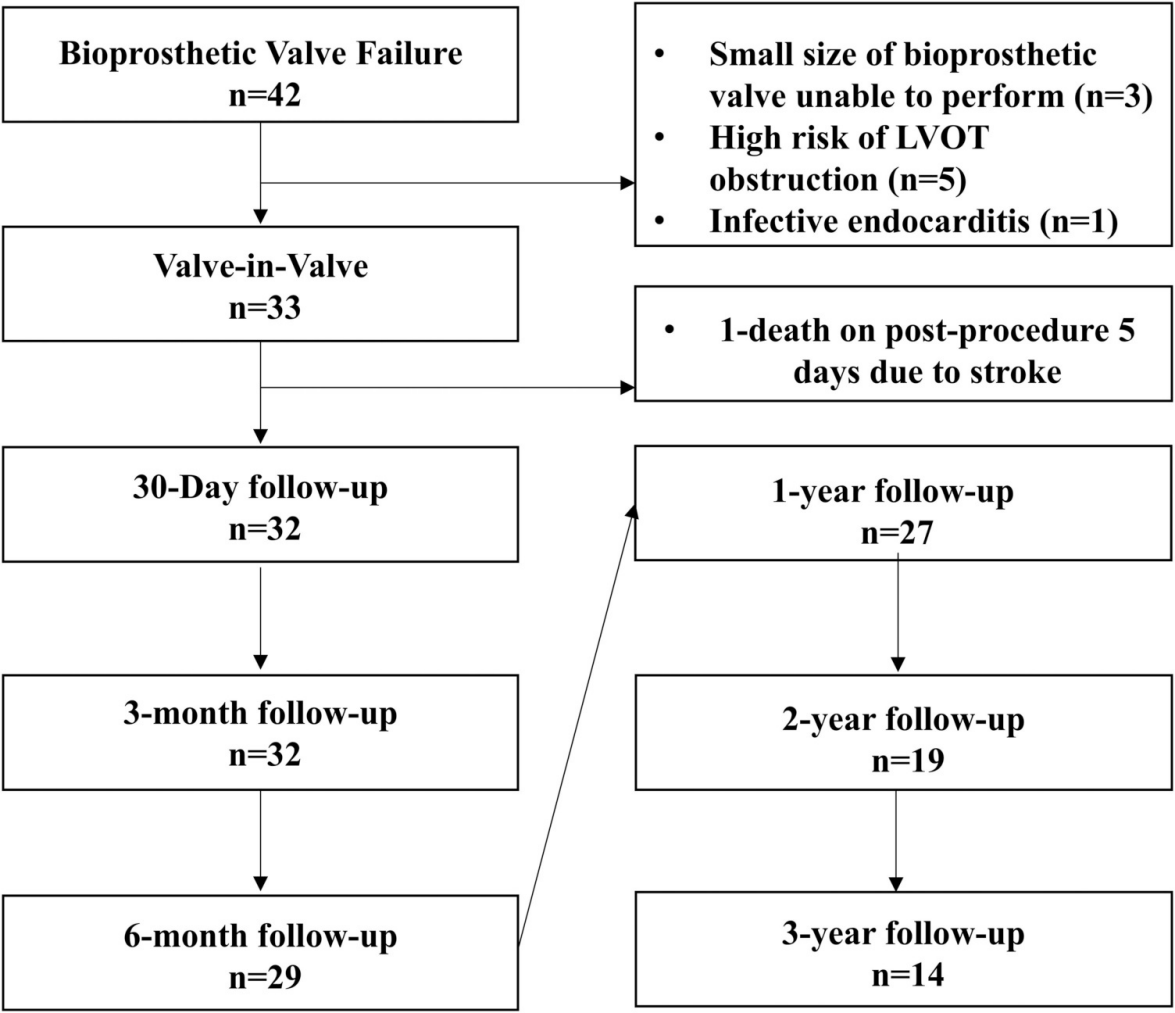
Enrolled patients and their follow-up.

This study was approved by the Ethics Committee of the First Affiliated Hospital of Zhengzhou University (L2021Q164). The data of all patients with biological valve dysfunction after mitral valve replacement who underwent TMVR surgery in our hospital from Jan 2019 to Jan 2024 were retrospectively collected. The collected data included echocardiography data, the New York Heart Association (NYHA) grade, the EuroQol visual analogue scale (EQ-VAS) score and the 6 min walking distance. According to the inclusion and exclusion criteria, 33 patients were ultimately included (Figure 1). The baseline characteristics of the patients are detailed in Table 1.

**Table 1:** Baseline characteristics.

Age(yrs)	70.70 ± 9.04
Male/ Female	8/25
Mitral valve replacement time (yrs)	11.67 ± 4.19
Mitral valve type and size	
Epic(25/27/29)	(8/10/3)
Medtronic Hancook II(25/27/29)	(7/1/1)
Edwards Perimount (25/27/29)	(1/1/1)
Type of mitral valve failure	
Severe insufficiency	14
Moderate insufficiency	4
Severe stenosis	4
Moderate stenosis	1
Stenosis combined with insufficiency	8
STS	10.36 ± 2.47
NYHA functional class	
I	0
II	0
III	15
IV	18
Multimorbidity	
Diabetes	10
COPD	15
Hypertension	20
Atrial fibrillation	18
Stroke	9
Coronary artery bypass	5
Aortic valve replacement	6
Tricuspid regurgitation	19

### Inclusion criteria

Previous artificial biological mitral valve replacement had led to valve dysfunction and required treatment; and before the operation, the second surgical valve surgery was classified as medium or high risk or contraindicated (STS score ≥6 points), or it was decided that conventional surgical operation was not suitable after discussion by the independent expert committee. The patients and their family members signed written informed consent forms.

### Exclusion criteria

Small-sized destructive bioprostheses that were not suitable for valve-in-valve treatment; or the assessment of mitral valve dysfunction, which indicates a high risk of severe left ventricular outflow tract obstruction (LVOTO) after mid-valve surgery. A Neo-LVOT area less than 1.8 cm^2^ is considered a contraindication for TMVR. Contraindications for cardiac catheterization surgery include infective endocarditis. Causing active infective endocarditis is an infectiological contraindication to transcatheter valve implantation due to risks of persistent bacteremia, periannular complications, and poor outcomes. After treated IE, residual vegetations/masses on the prosthesis can protrude into the LVOT and cause outflow obstruction (7, 8). Other contraindications including significant paravalvular leakage causing moderate to severe regurgitation; and other surgical operations, such as thoracotomy, that needed to be performed simultaneously.

All patients underwent transthoracic echocardiography (TTE) before the operation to determine the cause and degree of valvular lesions and whether they were combined with other valvular lesions. Computed tomography angiography of the aorta and coronary arteries was performed to determine the true inner diameter of the dysfunctional biological valve and whether there was a combination of coronary heart disease and macrovascular disease. The appropriate type and size of the valve were selected on the basis of the examination results.

## Procedure process

### Transapical approach

**Figure 2:**
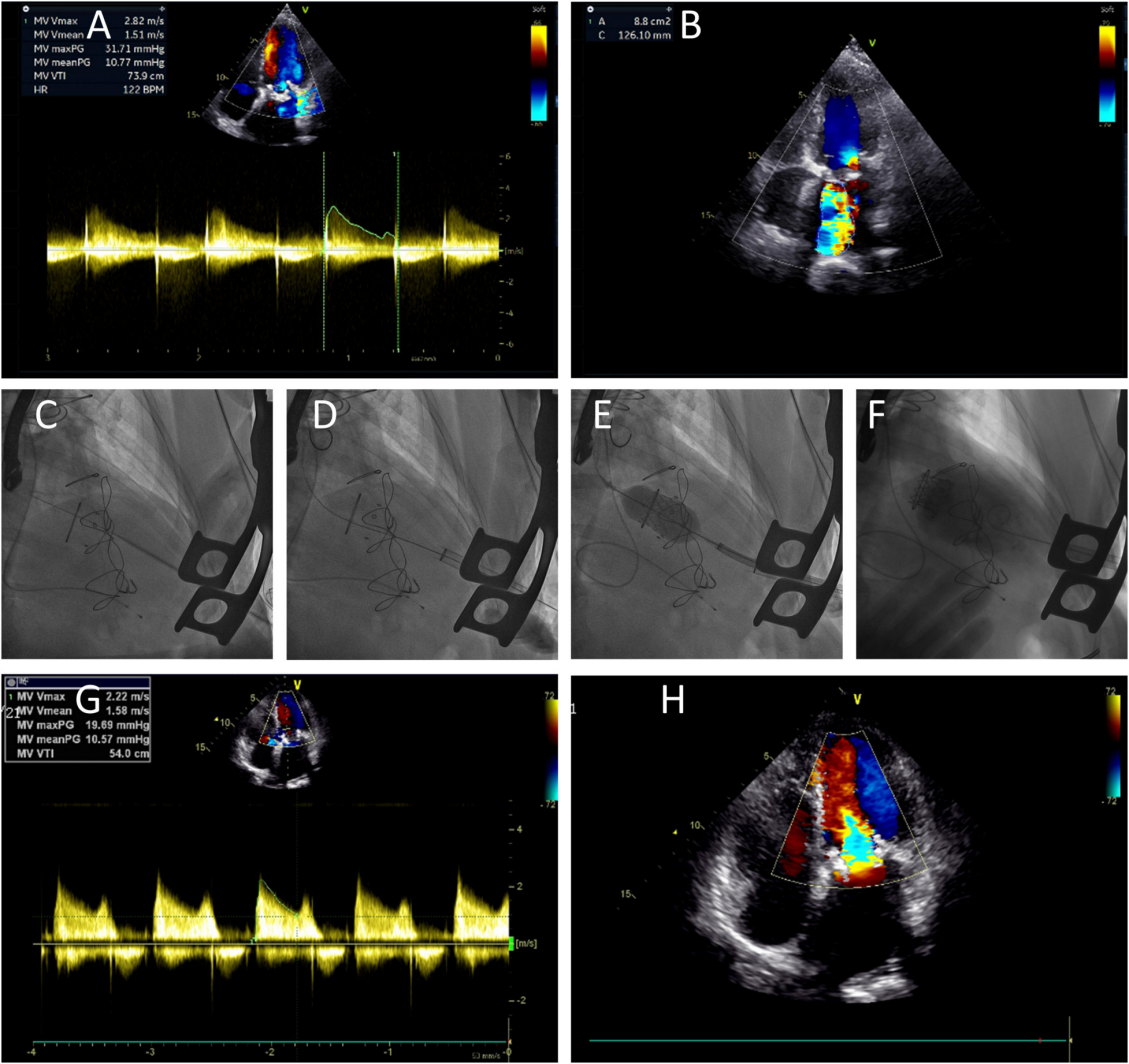
TMVR via the apical approach. Ultrasound revealed severe stenosis of the mitral valve biological valve **(A)** accompanied by mitral insufficiency **(B)** before the operation. The process of TMVR **(C–F)**. Postoperative ultrasound revealed a decrease in mitral valve pressure **(G)** and no regurgitation **(H)** after TMVR surgery.

A temporary pacing electrode was placed in the right internal jugular vein. The apical position was located under fluoroscopy. The fifth or sixth intercostal space on the left side was used to enter the chest. The apex of the suspended pericardium was exposed and heparinized at 1.5 mg/kg, and the activated whole blood coagulation time (ACT) was maintained at 250–300 s. Two rounds of suturing of the pericardium pouch were performed with 2–0 Prolene thread and felt pieces. Puncture was performed in the pouch area, the delivery sheath was introduced, the inner core was removed, and the valve was passed through the guide wire catheter to enter the left atrium, where the preshaped suprastiff guide wire was replaced. The valve was transported to the original dysfunctional mitral valve through the delivery system. The valve was gradually released. The delivery system was removed, and a porcine tail catheter was introduced. Left ventricular angiography and ultrasound confirmed good positioning. The peak flow velocity of the mitral valve and the average transvalvular pressure decreased, and there was no residual shunt. The conveyor sheath was removed. Protamine was used to neutralize heparin at a ratio of 1:1, the drainage tube was placed, the bleeding was stopped, and the chest was closed, which completed the operation (Figure 2).

### Transatrial septal approach

**Figure 3:**
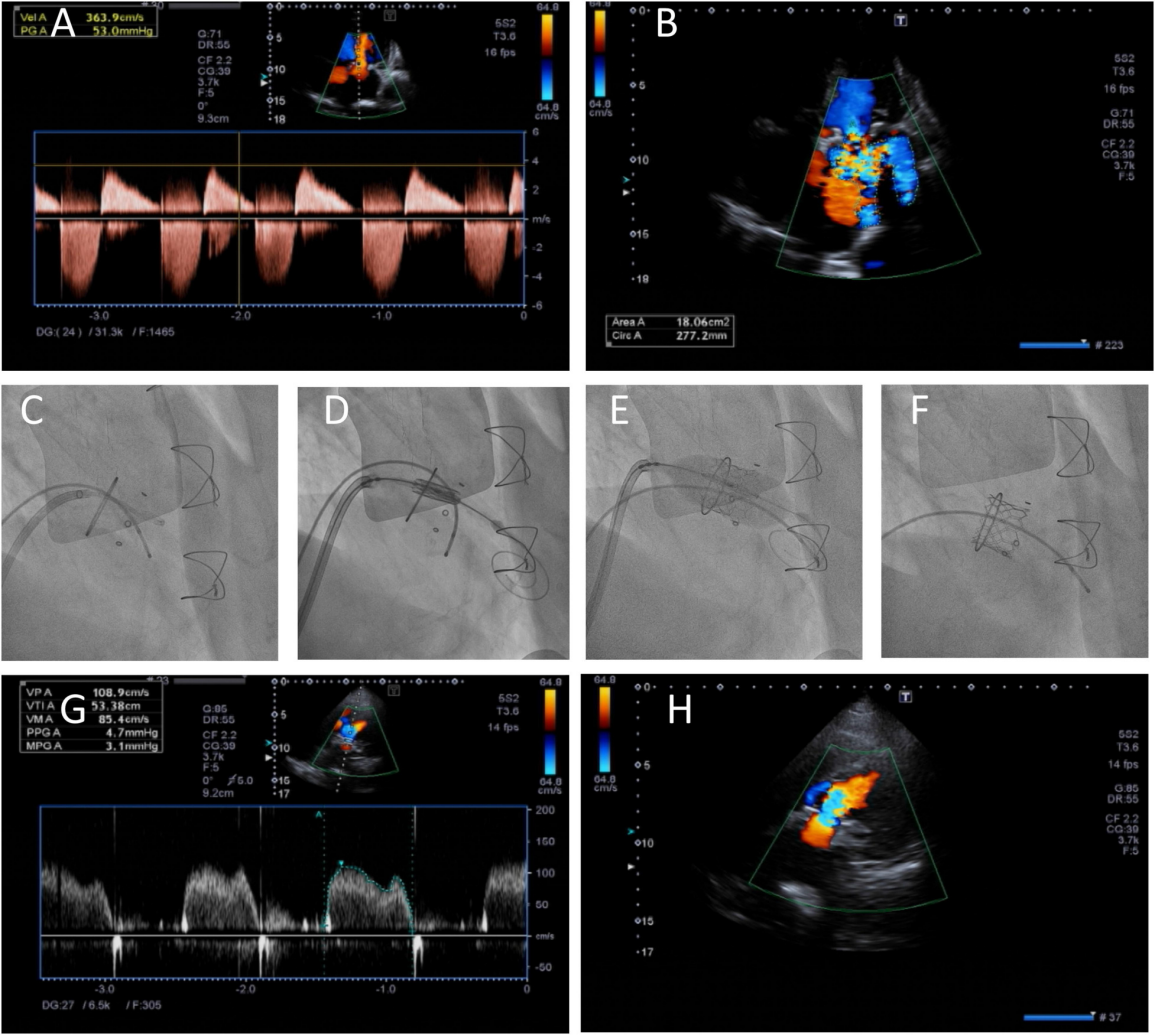
TMVR via the transseptal approach. Ultrasound revealed severe stenosis of the mitral valve biological valve **(A)** accompanied by mitral insufficiency **(B)** before the operation. The process of TMVR **(C–F)**. Postoperative ultrasound revealed a decrease in mitral valve pressure **(G)** and no regurgitation **(H)** after TMVR surgery.

Under ultrasound guidance, the left femoral artery was punctured for left ventricular angiography. A temporary pacing electrode was inserted into the left femoral vein. The right femoral vein was punctured, and the sheath was successfully placed. The atrial septal puncture guide wire was inserted through the sheath. The sheath was removed, and the Swartz sheath was inserted into the superior vena cava under the guidance of the guide wire. The guide wire was removed, the atrial septal puncture needle was inserted into the Swartz sheath, and the sheath was retracted to the oval fossa. After successful atrial septal puncture under ultrasound guidance, the puncture needle was removed, and the guide wire was introduced through the Swartz sheath. The Agilis sheath was replaced, the direction of the guidewire to the mitral valve orifice was adjusted, the guidewire was inserted along the guidewire into the pig-tail catheter, the mitral valve was crossed to the left ventricle, the ventricular guidewire was replaced, a balloon was inserted into the atrial septal puncture site for predilation, the balloon was withdrawn, the valve was inserted, and the valve was slowly released. The position was confirmed to be good by ultrasound, and there was no residual shunt. Protamine was used to neutralize the heparin, and the delivery system was removed, which completed the operation (Figure 3).

## Follow-up

All patients underwent ultrasound examinations 1 month, 3 months, and 6 months after the operation, and yearly examinations were performed as planned and followed up via phone calls when necessary.

## Statistical analysis

Figures were generated with GraphPad Prism 9 software. Continuous variables are summarized as mean ± standard. Whenever the variable is not normally distributed, the median and inter-quartile range (IQR) are reported instead. Categorical variables are presented as frequencies and percentages. Continuous variables were compared using Student's t-test or Mann–Whitney *U* test, depending on normality. Differences in pre-operation and follow-up data were analysed by analysis of variance. *post hoc* comparisons were performed via the Bonferroni method. Fisher's exact test and the chi-square test were used to compare categorical variables. All *P* values were 2-sided, and values < 0.05 were considered to indicate statistical significance. The statistical analysis was performed via SPSS 21.0.

## Results

A total of 33 patients were included in this study, including 8 males and 25 females, with an average age of 70.70 ± 9.04 years. The average age of the artificial biological mitral valve was 11.67 ± 4.19 years. There were 15 patients with a cardiac function classification of Grade III and 18 patients with Grade IV according to the New York Heart Association (NYHA). The multimorbidities included diabetes (in 10 patients), COPD (15 patients), hypertension (25 patients), atrial fibrillation (18 patients), stroke (9 patients), tricuspid regurgitation (19 patients), coronary artery bypass history (5 patients), and aortic valve replacement history (6 patients) (Table 1).

The procedural success defined according to Valve Academic Research Consortium-3 (VARC-3) criteria (9). All the 33 patients underwent TMVR surgery successfully. Seven patients underwent surgery via the atrial septal approach, and 26 patients underwent surgery via the apical approach. There was no valve displacement or secondary valve implantation. One patient experienced haemorrhage at the apical access site and underwent a second operation for haemostasis. Thus, the success rate of valve implantation was 97%. An Edwards SAPIEN3 valve was implanted in 7 patients, a J-valve was implanted in 14 patients, and a Renato VIV was implanted in 12 patients. Analysis of procedure-related metrics revealed that the transseptal group had a significantly longer operative time compared to the transapical group (179.43 ± 47.88 h vs. 130.19 ± 67.13 h, *P* = 0.046). The distribution of prosthetic valve types used differed significantly between the groups (*P* < 0.001). Regarding postoperative recovery outcomes, no statistically significant differences were observed between the groups for transfusion rates (16 vs. 2 patients, *P* = 0.203), postoperative hospital length of stay (10.15 ± 3.72 days vs. 10.00 ± 6.63 days, *P* = 0.936), ICU stay duration (51 IQRs: 20.65;62.85 h vs. 25.73 IQRs: 7.30;25.10 h, *P* = 0.538), or duration of intubation (37.9 IQRs: 23.00;50.10 h vs. 49.97 IQRs: 2.80;71.50 h, *P* = 0.261). The incidence of complications also showed no significant difference (*P* = 1.000). The transapical group experienced 5 complications, including one case each of hemorrhage, stroke, and death, along with two cases of pneumonia. The transseptal group had one case of pneumonia. The single mortality case occurred in the transapical group (Table 2). The VARC-3 procedural and early safety outcomes were shown in Table 3.

**Table 2:** Operation characteristics.

Access	Transapical	Transseptal	*P* value
Patient (No.)	26 (6/20)	7 (2/5)	0.763
Operation time (min)	130.19 ± 67.13	179.43 ± 47.88	0.046
Mitral valve type			<0.001
Renato (23/25/27)	11 (6/4/1)	1 (0/0/1)	
Edwards SAPIEN 3 (23/26)	1 (0/1)	6 (4/2)	
J-Valve(23/25/27)	14 (5/6/3)	0	
Blood transfusion	16	2	0.203
2–4 units RBC	11	1	
6 units RBC	5	1	
Hospital stays after operation (d)	10.15 ± 3.72	10.00 ± 6.63	0.936
ICU stays (h)	51	25.73	0.538
	(IQRs: 20.65;62.85)	(IQRs: 7.30;25.10)	
Intubation (h)	37.90	49.97	0.261
	(IQRs: 23.00;50.10)	(IQRs: 2.80;71.50)	
Complications	5	1	1
Hemorrhage	1	0	
Stroke	1	0	
Pneumonia	2	1	
Death	1	0	

**Table 3:** VARC-3 procedural and early safety outcomes.

Categories	Numbers
Valve implantation success	32/33
Type 3–4 bleeding	1/33
Stroke	1/33
Acute kidney injury	0/33
30-day mortality	1/33

The follow-up diagram is shown in Figure 1. All 32 patients completed at least 3 months of follow-up. Nineteen patients completed 6 months, 17 completed 1 year, 19 completed 2 years, and 14 completed 3 years of follow-up. At the 2-year follow-up, only 1 patient had died, with the other 31 patients still surviving.

**Figure 4:**
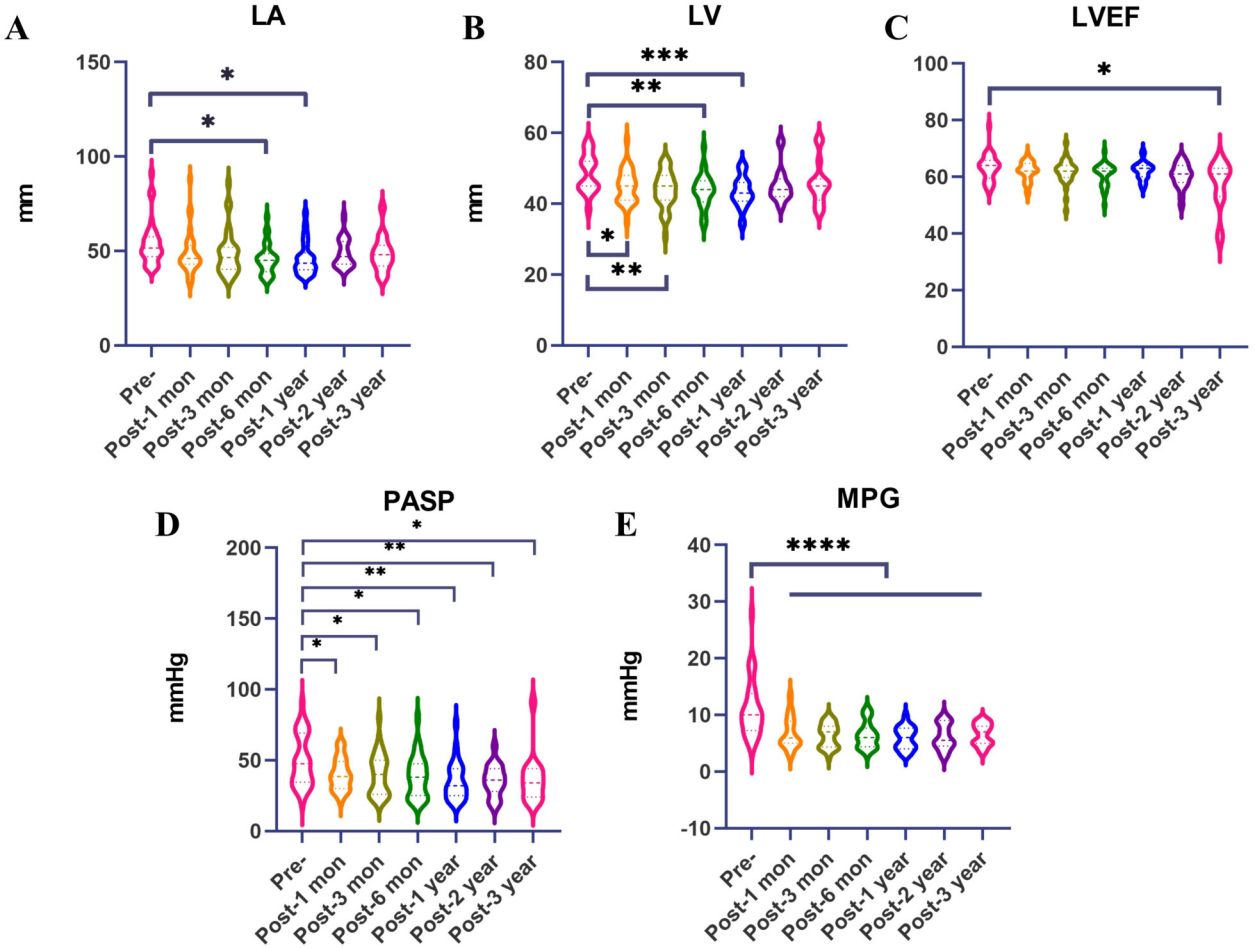
Comparative analysis of echocardiography data before and after surgery. Left atrium (LA) diameter **(A)**; left ventricle (LV) diameter **(B)**; left ventricular ejection fraction (LVEF) **(C)**; pulmonary artery systolic pressure (PASP) **(D)**; mitral pressure gradient (MPG) **(E)**. *P* values < 0.05 (*), <0.01 (**), <0.001 (***), and <0.0001 (****).

**Figure 5:**
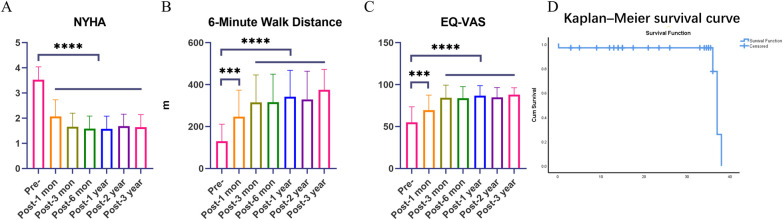
Comparative analysis of the NYHA grade **(A)**, EQ-VAS score **(B)** and 6 min walking distance **(C)** and Kaplan–Meier survival curve **(D)**. *P* values < 0.05 (*), <0.01 (**), <0.001 (***), and <0.0001 (****).

The diameter of the left atrium decreased from 53.94 ± 11.59 to 45.79 ± 7.99 and 46.08 ± 8.64 at 6 months and 1 year after the surgery, respectively, and these differences were statistically significant (both *P* < 0.05). The diameter of the left ventricle decreased from 48.66 ± 5.37 to 44.84 ± 4.70, 43.97 ± 5.04, 43.69 ± 4.65, and 43.27 ± 4.29 at 1 month to 1 year postsurgery, and there were significant differences compared with presurgery values (all *P* < 0.05). The left ventricular ejection fraction (LVEF) decreased from 63.28 ± 4.59 to 57.40 ± 8.37 at 3 years postsurgery (*P* < 0.05). The pulmonary artery systolic pressure (PASP) decreased from 49.72 ± 17.85 mmHg to 40.16 ± 11.14 mmHg, 41.56 ± 20.87 mmHg, 38.55 ± 14.58 mmHg, 35.42 ± 13.22 mmHg, 35.68 ± 10.82 mmHg, and 36.80 ± 16.89 mmHg from 1 month to 3 years postsurgery, and these values were significantly different from those before surgery (all *P* < 0.05). The pressure gradient (MPG) decreased from 11.07 ± 5.02 mmHg to 6.81 ± 2.77 mmHg, 6.38 ± 2.04 mmHg, 6.44 ± 2.25 mmHg, 5.93 ± 1.94 mmHg, 6.20 ± 2.21 mmHg, and 6.56 ± 1.83 mmHg from 1 month to 3 years postsurgery, and there were significant differences compared with presurgery values (all *P* < 0.05) (Figure 4). Compared with those before surgery, the NYHA grade (Figure 5A), EQ-VAS score (Figure 5B) and 6 min walking distance improved significantly at follow-up (all *P* < 0.05) (Figure 5C). The Kaplan–Meier survival curve was shown in Figure 5D.

## Discussion

The use of TMVR has become more common in the treatment of mitral bioprosthetic valve dysfunction. This study revealed that (1) the mitral valve-in valve was associated with a lower 30-day mortality than predicted by the Society of Thoracic Surgeons score; (2) the mitral valve-in valve was associated with sustained improvement in functional class and 6 min walk distance, improved quality-of-life scores and significantly diminished symptoms at follow-up; and (3) patients receiving the mitral valve-in valve had excellent survival at follow-up.

Unlike prior retrospective studies that reported that the use of the mitral valve-in valve is feasible but associated with procedural complications and high 30-day and 1-year mortality rates (10), our results revealed that the use of the mitral valve-in valve is safer and more efficient. The 30-day and 1-year mortality rates in the entire cohort were 3%, which were lower than those reported in prior studies. In the study of Alvarez-Covarrubias (11), a total of 56 patients received valve in valve TMVI, the 30-day and 1-year mortality was 10.7% and 23.2% respectively. Furthermore, another large number registry-based prospective cohort study of Whisenant (12) reported the all-cause mortality was 5.4% at 30 days and 16.7% at 1 year. The small number cohort may explain the discrepancy between our study and what has been reported. These findings are similar to the results of the prospective study conducted by Mayra and colleagues (5). Only one case was associated with procedure-related haemorrhage, and one patient experienced cerebral infarction complications and eventually died 7 days after the operation.

LVOTO is a potentially serious complication in transcatheter valve surgery. The incidence rate of LVOTO is approximately 3.2% (13). In cases of LVOTO, typically the inclination angle of the blood flow entering the mitral valve was too large, or the implantation position was not optimal, resulting in forward systolic movement of the anterior lobe of the mitral valve (14, 15). However, no LVOTO complications occurred in our study. The main reason lies in the fact that we conducted thorough valve evaluations before and during the operation. Generally, excessive penetration of the valve into the ventricle should be avoided to reduce the potential impact on the left ventricular outflow tract. For some people at high risk of obstruction, anterior mitral valve tear surgery can be performed when necessary to effectively prevent the occurrence of LVOTO. The occurrence of this complication was predicted by evaluating the area of the new left ventricular outflow tract through multiplanar reconstruction of cardiac CT before the operation. During the operation, the optimal implantation position was determined according to the model of the surgical valve, which can effectively reduce the occurrence of LVOTO.

Among the patients included in this study, 2 had mild perivalvular leakage after the operation. The peak mitral valve flow velocity of the other 5 patients did not improve significantly immediately after the operation (≥2.5 m/s), and the difference in transvalvular pressure in 2 patients was greater than 10 mm Hg after the operation. During interventional mitral valve surgery, owing to the constraint of the original mitral biological valvular annulus, incomplete dilation of the interventional valve may also lead to poor valve function, a high residual pressure difference and early structural damage to the valve. Cheung et al. (16) reported that the average pressure difference at discharge decreased from 11.1 ± 4.6 mmHg to 6.9 ± 2.2 mmHg in 23 consecutive patients who underwent mitral valve implantation. However, the average transvalvular pressure difference was 9 mmHg in 2 patients and 10 mmHg in 2 patients. The average pressure difference of one patient in the study by Seiffert et al. (17) was 12 mmHg. The average pressure difference of one patient in the study by Wilbring et al. (18) was 9 mmHg. Similarly, in this study, although the symptoms of 2 patients were significantly relieved, the average transvalvular pressure difference after the operation still exceeded 10 mmHg. The analysis suggests that this might be due to the patients' smaller body size and the selection of a smaller valve model in the first surgical mitral valve bivalve replacement, resulting in residual pressure differences remaining after the interventional implantation of the valve.

There are several limitations in this study. First, this was a retrospective study with a small number of patients enrolled and was not compared with other surgeries, such as valve-in-ring and valve-in-MAC. Prospective randomized controlled studies are needed for further verification. Second, owing to the small number of included patients, this study conducted a comprehensive analysis of the apical approach and the femoral vein approach, without further comparative analysis. Third, although this study included three years of follow-up, a longer follow-up period is still needed to confirm its effectiveness.

## Conclusion

Valve-in-valve TMVR is a feasible option for patients with degenerated bioprosthetic mitral valves. However, follow-up studies are still needed to determine the long-term effects.

## Data Availability

The original contributions presented in the study are included in the article/Supplementary Material, further inquiries can be directed to the corresponding author.
